# Psoriasis as a systemic inflammatory disease: an immune set-point framework for comorbidities and relapse

**DOI:** 10.3389/fimmu.2026.1830099

**Published:** 2026-05-15

**Authors:** Jingyi Ma, Haoyu Li, Xueqi Yang, Yuzhe Cheng, Bingran Qi, Siyu Kang, He Qi, Ruiqun Qi, Jun Niu

**Affiliations:** 1 Department of Dermatology, General Hospital of Northern Theatre Command, Shenyang, Liaoning, China; 2 Department of Dermatology, The First Hospital of China Medical University, Shenyang, China; 3 Key Laboratory of Immunodermatology, Ministry of Education, and National Health Commission, National Joint Engineering Research Center for Theranostics of Immunological Skin Diseases, Shenyang, China

**Keywords:** comorbidities, immune set-point, psoriasis, relapse, systemic inflammation

## Abstract

Psoriasis is increasingly recognized as a systemic immune-mediated disorder that extends beyond cutaneous inflammation. Although skin lesions are its most visible manifestation, growing evidence indicates that psoriasis reflects a broader disruption of immune homeostasis accompanied by sustained systemic inflammatory activation. Persistent immune activation, altered myeloid programming, and dysregulated immunometabolic pathways together establish a state of chronic inflammatory priming. This systemic inflammatory state contributes to multi-organ comorbidities and also favors disease relapse through durable immune memory and trained immunity. In this review, we propose that psoriasis reflects maladaptive reprogramming of the systemic immune set-point, such that both spatial multi-organ involvement and temporal disease recurrence arise from shared immunologic mechanisms. Viewing psoriasis in these terms has important implications for comorbidity assessment, long-term maintenance strategies, and the pursuit of disease modification.

## 1 Introduction

Psoriasis is increasingly recognized as a systemic immune-mediated disorder, not merely a condition confined to cutaneous inflammation. Although psoriatic plaques are the most visible clinical manifestation, converging epidemiologic, translational, and mechanistic evidence indicates that the underlying immune disturbance extends beyond lesional skin (1). Circulating inflammatory mediators, bone marrow activation, vascular inflammation, and metabolic dysregulation all point to a broader disruption of immune homeostasis (2, 3).

Psoriasis also imposes a substantial global health burden. Recent estimates suggest that approximately 43 million people worldwide were living with psoriasis in 2021, with reported prevalence varying across regions (4). Although prevalence appears higher in Europe, North America, and Australasia than in parts of Asia and sub-Saharan Africa, these comparisons remain constrained by incomplete epidemiologic coverage and probable under-ascertainment in many settings (5).

At the clinical level, several observations suggest that skin activity alone does not fully capture the biological scope of psoriasis. Systemic inflammatory signatures may be detected even in some patients with limited skin involvement, and cardiometabolic risk does not consistently parallel lesion extent. Disease recurrence also cannot always be explained by purely local reactivation. Jointly, these findings point to a more persistent state of immune activation that extends beyond visible plaques.

Emerging evidence also supports a model in which impaired immune tolerance and sustained activation of systemic immune responses establish a state of chronic inflammatory priming (6). In plaque psoriasis, regulatory T-cell dysfunction, enhanced dendritic cell activation, and heightened responsiveness of the IL-23–IL-17 pathway weaken mechanisms that normally restrain inflammatory amplification (7, 8). Beyond the skin, metabolic reprogramming and functional reconfiguration of both myeloid and lymphoid populations have also been described, indicating that innate and adaptive compartments jointly contribute to systemic immune activation (9–11). These changes are more consistent with persistent elevation of the systemic immune set-point than with a transient episode of inflammatory overactivation.

This broader immune milieu offers a plausible explanation for multi-organ involvement. Elevated circulating cytokines exert functional effects on vascular endothelium, adipose tissue, and hematopoietic niches, supporting the idea of interconnected inflammatory circuits (12, 13). Molecular imaging studies reinforce this perspective: FDG-PET/CT demonstrates increased metabolic activity in hematopoietic organs that parallels vascular inflammation and subclinical atherosclerosis, consistent with an integrated cardio-hematopoietic inflammatory axis in psoriasis (14, 15). In this sense, cutaneous plaques may be viewed as the visible organ-level expression of a more pervasive systemic immune state.

The temporal dimension of recurrence provides further insight into psoriasis pathophysiology. Even after clinical resolution, previously involved skin sites retain tissue-resident memory T cells capable of rapidly reigniting local inflammation in response to environmental or endogenous triggers (7). At the same time, innate immune cells in psoriasis display features of trained immunity, characterized by persistent metabolic and epigenetic reprogramming that heightens responsiveness to inflammatory stimuli (16, 17). Collectively, these adaptive and innate memory mechanisms create a permissive immunologic landscape that favors relapse after apparent disease clearance.

Psoriasis also spans a spectrum with both autoimmune and autoinflammatory features. Chronic plaque psoriasis is characterized by reciprocal interplay between adaptive Th17 responses and innate amplification pathways (1). Generalized pustular psoriasis (GPP), by contrast, is frequently associated with dysregulation of the IL-36 pathway and is marked by prominent neutrophilic inflammation and systemic manifestations (18). These phenotypes are not necessarily separate disease entities. They may instead represent different expressions of a shared framework of systemic immune dysregulation, with varying contributions from adaptive and innate immune pathways.

In this review, we propose that psoriasis reflects maladaptive reprogramming of the systemic immune set-point. Within this framework, both spatial multi-organ involvement and temporal disease recurrence arise from shared immunologic mechanisms, including breakdown of immune tolerance, chronic inflammatory priming, and durable immune memory. Viewing psoriasis in these terms has important implications for comorbidity assessment, long-term maintenance strategies, and the pursuit of disease modification.

## 2 Systemic immune set-point dysregulation: a conceptual framework for psoriasis

The initiation and resolution of immune responses are tightly regulated by multiple checkpoints that concurrently maintain immune tolerance and tissue homeostasis (19). Under physiological conditions, antigen exposure triggers an orchestrated cascade involving antigen presentation, activation of adaptive immune responses and recruitment of effector cells to peripheral tissues. Once the inciting stimulus has been eliminated, regulatory mechanisms—including central and peripheral tolerance, inhibitory signaling pathways and tissue-specific regulatory circuits—act to terminate inflammation and restore immune equilibrium (20, 21).

Psoriasis arises when these regulatory mechanisms become destabilized in genetically susceptible individuals exposed to environmental perturbations. Genome-wide association studies have identified numerous susceptibility loci linked to antigen presentation, cytokine signaling and immune regulation, indicating that host genetic background helps shape the regulatory landscape within which the immune set-point is established (22). Genetic predisposition alone, however, is not sufficient to initiate disease.

Current models of autoimmune skin disease suggest that psoriasis develops through the accumulation of stochastic perturbations across multiple levels of immune regulation (23). Within this multi-hit framework, environmental stimuli—including infection, mechanical injury, metabolic stress, psychological stress and pharmacological exposures—introduce sequential disruptions at key checkpoints such as antigen exposure, antigen presentation, lymphocyte activation and inflammatory resolution. Rather than producing a single transient inflammatory episode, these cumulative insults progressively destabilize immune regulatory networks.

Importantly, inflammatory diseases rarely arise from dysfunction in a single cell type or signaling pathway. More often, they reflect coordinated alterations across interconnected immune circuits (24). In psoriasis, innate immune activation, antigen presentation and adaptive immune responses converge to generate a self-reinforcing inflammatory network centered on the IL-23–T17 axis (1). Activated dendritic cells, neutrophils and macrophages produce cytokines such as IL-23, IL-1β and TNF, which promote the differentiation and stabilization of IL-17-producing lymphocyte populations, including Th17 cells, Tc17 cells and γδ T cells (25–27). Additional immune populations, including Th22 cells, Th9 cells, and innate lymphoid cells, also contribute to psoriatic inflammation, underscoring the complexity of the broader inflammatory network underlying disease pathogenesis (28). Once established, this feed-forward inflammatory circuit becomes at least partially independent of the initiating trigger. Stromal cells interact reciprocally with activated immune cells, creating a positive feedback loop that sustains local inflammation and promotes inflammatory memory (29, 30). In this way, psoriasis is not maintained by immune cells alone. It is stabilized through ongoing interactions between immune and structural compartments, with the potential to extend beyond the skin and engage broader systemic responses (31).

**Figure 1:**
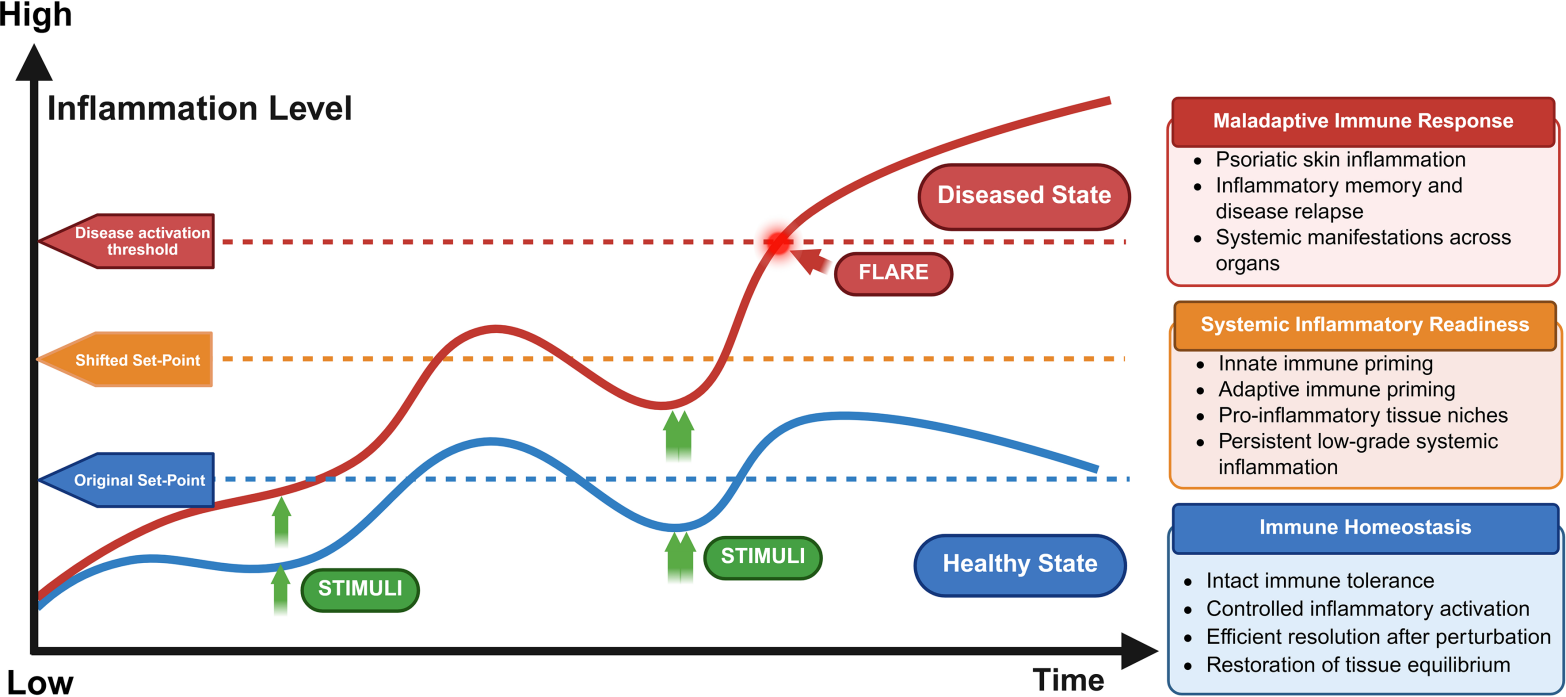
Threshold model of systemic immune set-point dysregulation in psoriasis. This image illustrates psoriasis as a threshold disorder in which cumulative inflammatory perturbations shift the systemic immune set-point toward a persistently primed state closer to the threshold for overt inflammatory activation. Created with BioRender.com.

Here, we use the term immune set-point to describe the basal inflammatory operating state of the immune system that determines how close it resides to the threshold for inflammatory activation and how readily it returns to homeostasis after perturbation. Within this framework, psoriasis may first become clinically apparent in the skin when a persistently primed immune state crosses the threshold for overt inflammatory expression, while already reflecting a broader network-level disorder of immune regulation (**Figure 1**).

## 3 Systemic inflammatory burden

If psoriasis is viewed as a disorder of systemic immune set-point dysregulation, the next question is how this altered immune state becomes apparent beyond the skin. One major consequence is the emergence of a persistent systemic inflammatory burden.

### 3.1 Systemic inflammatory burden as a durable, multi-compartment adaptation

Within this framework, systemic inflammatory burden is better understood as a durable, multi-compartment adaptation than as a transient spillover from localized cutaneous inflammation. This view is consistent with the broad genetic architecture of psoriasis. Susceptibility loci implicate not only immune pathways but also keratinocyte-intrinsic programs, including skin-barrier genes such as LCE3, KLF4, and CDSN (32). Disease-relevant reprogramming therefore appears to extend across both immune and structural compartments.

One measurable dimension of systemic inflammatory burden lies in altered activity within circulating innate immune compartments. Peripheral blood transcriptomic studies in psoriasis have identified inflammatory signatures enriched for neutrophil- and NK cell-associated pathways (33), pointing to systemic activation of myeloid programs that is not fully captured by skin-limited disease metrics. Neutrophils, which originate in the bone marrow and are recruited to inflamed tissues, accumulate in psoriatic lesions and form characteristic histopathologic structures such as Munro’s microabscesses and the spongiform pustules of Kogoj. Systemic myeloid mobilization is therefore directly linked to canonical tissue pathology. Experimental work further suggests that psoriatic inflammatory cues can induce durable functional reprogramming in myeloid cells. For example, LL37–self-DNA complexes have been shown to trigger metabolic and epigenetic remodeling in monocytes consistent with trained immunity-like priming (34).

A second component of systemic inflammatory burden involves persistent alterations in adaptive immune circuits. Psoriasis is sustained by type 17-skewed lymphocyte responses downstream of IL-23. Recent single-cell and interventional studies support a model in which IL-23–T17-centered immune responses can be attenuated by therapy but are not uniformly reset across different immune compartments (35–37). Although the extent to which antigen-recognition thresholds are broadly altered across patients remains uncertain, single-cell transcriptomic and TCR profiling studies consistently identify clonally skewed and functionally specialized T17 cell states in both skin and synovial tissue, pointing to an adaptive immune landscape that is more readily reactivated (38–40).

The systemic inflammatory landscape is further reinforced by stromal cells within affected tissues. Keratinocytes actively amplify inflammatory signaling through the production of antimicrobial peptides, cytokines, and chemokines that promote leukocyte recruitment and effector polarization (41, 42). Dermal fibroblasts and endothelial cells contribute in parallel by regulating leukocyte trafficking, vascular activation, and tissue remodeling (41, 43). Through these reciprocal interactions, immune–stromal niches emerge that sustain local immune responses, promote the transition of effector cells toward memory states, and reinforce broader inflammatory circuits through type 3 immune-mediated feedback.

Durable metabolic and epigenetic cellular states may further stabilize this inflammatory milieu. Systemic metabolic perturbations, including obesity-associated changes in lipid and nutrient availability, can reshape immunometabolic processes across both myeloid and lymphoid lineages and promote sustained Th17 effector activity (44). At the tissue level, metabolic programs within the skin epithelium closely interact with type 17 immune responses (44, 45), highlighting how epithelial–immune metabolic coupling can amplify inflammatory circuits that extend from the skin into systemic immune compartments. Collectively, these adaptations provide a mechanistic basis for the emergence and persistence of systemic inflammatory burden in psoriasis.

### 3.2 Persistent low-grade systemic inflammation

Persistent abnormalities in circulating inflammatory mediators provide measurable evidence of systemic immune activation in psoriasis. Cytokines linked to the IL-23–T17 axis, including IL-17A, IL-23, and TNF, can be detected in the circulation and have been associated with disease severity, comorbidity burden, and response to biologic therapy (46–48). These mediators are unlikely to represent passive leakage from inflamed skin alone. More plausibly, they reflect activation of a broader type 3 immune network operating across multiple systemic compartments.

Chemokine pathways offer additional insight into how systemic immune activation is sustained through directed immune-cell trafficking. CCL20, the ligand for CCR6, is strongly induced in activated keratinocytes and secondary lymphoid tissues and plays a central role in the differentiation, recruitment, and skin homing of CCR6+ immune populations, including Th17 cells, γδ T cells, and dendritic cells. Elevated circulating levels of CCL20 have been reported in patients with psoriasis, consistent with dynamic communication between inflamed tissues and systemic immune compartments (49). In this setting, chemokine gradients do more than direct cell migration. They also help reinforce inflammatory circuits linking the skin, lymphoid organs, and peripheral blood.

Further evidence of systemic immune perturbation comes from inflammatory indices derived from routine laboratory parameters. Biomarkers such as C-reactive protein (CRP), the neutrophil-to-lymphocyte ratio (NLR), and the systemic immune-inflammation index (SII) integrate information from circulating leukocyte populations and acute-phase inflammatory responses. Elevated CRP has been associated with psoriasis severity and cardiometabolic comorbidity risk (50, 51), whereas NLR and SII have been proposed as surrogate markers of systemic myeloid activation and leukocyte redistribution (52, 53). GlycA, a more recently described marker of low-grade chronic systemic inflammation, has also been linked to psoriasis and related cardiovascular disease (54). Although none of these markers is specific to psoriasis, they provide accessible indicators of the broader inflammatory burden accompanying systemic immune activation.

At the same time, systemic inflammatory markers are not uniformly elevated across all patients. Population studies suggest substantial heterogeneity in inflammatory profiles, with some individuals showing pronounced systemic cytokine signatures while others exhibit relatively limited systemic abnormalities despite active skin disease (55). This variability likely reflects differences in underlying immunologic pathways, disease stage, and comorbidity burden. Circulating biomarkers may be most useful when viewed as reflecting distinct inflammatory phenotypes, not simply as universal indicators of disease presence.

Overall, these observations support the view that psoriasis is accompanied by a sustained systemic inflammatory burden that extends beyond the skin. This burden is maintained through the interaction of several processes, including systemic myeloid activation with trained immunity-like priming, persistent or clonally biased type 17 adaptive immune responses, inflammatory amplification by structural cells, and durable metabolic and epigenetic changes across immune compartments. Altogether, these adaptations provide a cellular and molecular basis for systemic immune set-point dysregulation and help explain the persistence and systemic propagation of psoriatic inflammation.

## 4 Temporal consequences: inflammatory memory and disease relapse

**Figure 2:**
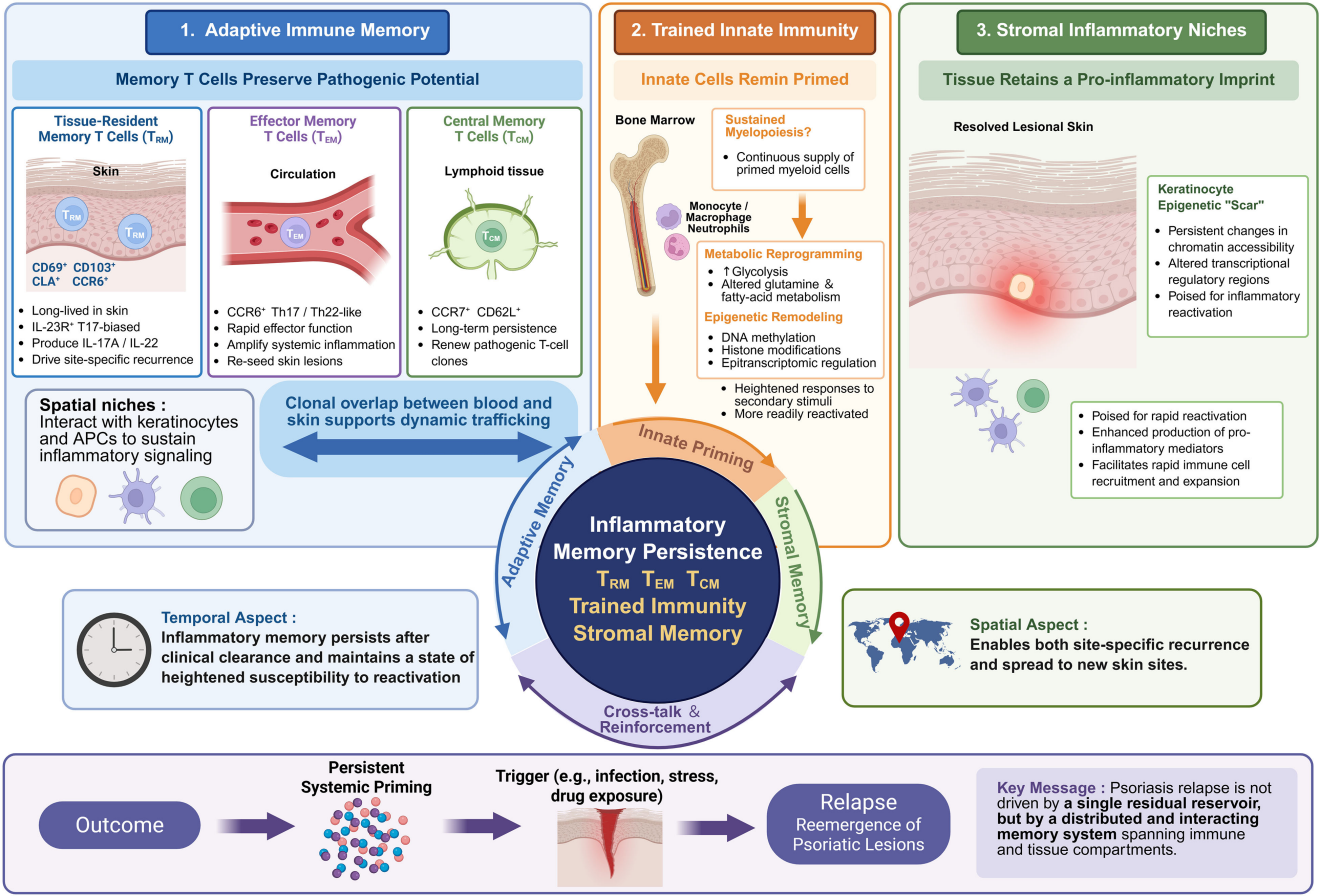
Distributed inflammatory memory as a driver of psoriasis relapse within the systemic immune set-point framework. This image summarizes how psoriasis relapse is sustained by distributed inflammatory memory across adaptive immune, innate immune, and stromal compartments. Viewed together, these interacting memory layers bring the system closer to the threshold for reactivation and support both site-specific recurrence and extension to new skin sites. APCs, antigen-presenting cells; CCR6, C-C motif chemokine receptor 6; CCR7, C-C motif chemokine receptor 7; CD, cluster of differentiation; CLA, cutaneous lymphocyte antigen; IL, interleukin; IL-23R, interleukin-23 receptor; TCM, central memory T cells; TEM, effector memory T cells; Th, T helper; TRM, tissue-resident memory T cells. Created with BioRender.com.

Beyond its multi-organ manifestations, systemic immune set-point dysregulation in psoriasis also has an important temporal consequence: disease relapse. Although biologic therapies targeting the TNF, IL-23 and IL-17 pathways can induce rapid clinical clearance, durable treatment-free remission remains uncommon, and relapse frequently follows treatment discontinuation (56). This clinical pattern has brought increasing attention to the concept of inflammatory memory. In its classical sense, immune memory refers to the capacity of adaptive immune cells to respond more rapidly upon antigen re-encounter (57). In chronic inflammatory diseases such as psoriasis, however, memory extends beyond its original protective role in host defense. Instead, persistent imprinting across immune and tissue compartments may sustain pathogenic inflammatory potential and predispose the disease to recur. As summarized in **Figure 2**, relapse and extra-cutaneous disease extension arise from persistent inflammatory memory operating across adaptive immune, innate immune, and stromal layers. From this perspective, the relapsing course of psoriasis reflects a state of ongoing inflammatory priming maintained by memory T-cell populations, trained innate immune states and stromal inflammatory niches.

### 4.1 Memory T cells

Memory T cells are generally viewed as the most convincing cellular explanation for psoriasis relapse. This idea has been strengthened by high-resolution approaches such as single-cell RNA sequencing, TCR repertoire analysis and spatial transcriptomics, which have made it possible to track pathogenic T-cell populations with much greater precision. A consistent observation has emerged from these studies: even when lesions appear clinically resolved, disease-associated T-cell clones can remain in the skin and retain the capacity to rekindle inflammation. Importantly, this residual inflammatory memory does not seem to reside in a single subset. Rather, it is distributed across several memory T-cell compartments, including tissue-resident memory T cells (TRM), effector memory T cells (TEM) and central memory T cells (TCM).

Among these, TRM cells have been studied most extensively in psoriasis. These long-lived lymphocytes reside in peripheral tissues over prolonged periods and are typically defined by tissue-retention markers such as CD69 and CD103, in concert with skin-homing molecules including cutaneous lymphocyte antigen (CLA) and C-C chemokine receptor type 6 (CCR6) (58). Because they remain in non-lymphoid tissue instead of recirculating continuously through the blood, TRM cells are well positioned to mount rapid local responses when inflammatory cues reappear.

In psoriatic skin, TRM cells are biased towards type 17 immunity and express receptors linked to IL-23 responsiveness, including IL-23R (7). Upon reactivation, both CD4^-^ and CD8^-^ TRM populations in the epidermis and dermis can produce IL-17A and IL-22, thereby promoting keratinocyte activation and reinforcing local inflammatory circuits (59). Spatial transcriptomic studies further suggest that these cells are not randomly distributed, but instead occupy localized immune niches within the epidermis, where they engage with keratinocytes and antigen-presenting cells to sustain inflammatory signaling (60). This is consistent with observations that psoriatic T17-associated transcriptional programs can still be detected in clinically resolved lesions, implying that pathogenic T-cell states persist despite apparent remission (35, 61). Such persistence offers a plausible mechanistic basis for one of the most characteristic features of psoriasis: the tendency of lesions to recur at the same anatomical sites.

That said, a TRM-centered view is not sufficient on its own. It explains why psoriasis often returns to previously affected skin, but it does not fully capture the broader heterogeneity of relapse. Clinically, flares are not always confined to old sites. New lesions may also develop in previously uninvolved areas, particularly in the setting of systemic triggers such as infection or drug exposure. This pattern argues against a purely local memory model and suggests that relapse also depends on immune mechanisms operating beyond the skin.

Circulating memory T cells reflect another important layer of pathogenic memory. TEM cells lack lymphoid-homing receptors such as CCR7 and CD62L and are equipped for immediate effector function, whereas TCM cells retain these receptors and provide longer-term proliferative capacity within lymphoid tissues (62). In psoriasis, circulating memory T-cell pools are skewed towards type 3 immune programs. Peripheral CCR6^-^ effector memory T cells are enriched for Th17 and Th22 like phenotypes, creating a mobile reservoir of pathogenic cells that may amplify systemic inflammation (63, 64).

Evidence from TCR repertoire studies adds further support to this view. Considerable clonal overlap has been observed between circulating T cells and skin-infiltrating lymphocytes, indicating ongoing trafficking between blood and skin compartments (38). This dynamic exchange raises the possibility that circulating memory T cells can reseed inflammatory lesions, while lymphoid TCM populations preserve the longer-term continuity of pathogenic T-cell clones.

Within the systemic immune set-point framework proposed in this review, these findings support a layered model of adaptive immune memory in psoriasis. TRM cells anchor inflammatory memory in previously affected skin and enable rapid local reactivation. TEM cells provide a circulating effector pool that can extend and amplify inflammation systemically. TCM cells, in turn, help sustain the long-term persistence and renewal of pathogenic clones. Relapse is better characterized as the output of a distributed memory system spanning skin, blood and lymphoid tissue.

### 4.2 Trained innate immunity

Adaptive immune memory is only part of the picture. Innate immune cells may also retain a form of inflammatory memory that contributes to relapse. This phenomenon, commonly referred to as trained immunity, describes the durable functional reprogramming of innate immune cells—especially monocytes and macrophages—after inflammatory stimulation, driven by coordinated metabolic and epigenetic changes (65).

Several features of psoriatic inflammation are compatible with this concept. Alterations in glycolysis, glutamine metabolism and fatty-acid metabolism, in conjunction with epigenetic mechanisms such as DNA methylation, histone modification and epitranscriptomic regulation, may reshape innate immune-cell behavior in a more persistent way than previously appreciated (11, 34, 66). In particular, psoriasis-associated inflammatory cues have been shown to induce metabolic and epigenetic remodeling in monocytes, supporting the existence of trained immunity-like priming in the disease setting (34). These findings suggest that innate immune cells in psoriasis may fail to return fully to a basal state after inflammation subsides and may instead remain predisposed to mount heightened responses to subsequent stimuli (67).

What remains less clear is how far this reprogramming extends and how directly it contributes to relapse. In other inflammatory disorders, inflammatory signals can reach the bone marrow niche and influence hematopoietic stem and progenitor cells, promoting sustained pro-inflammatory myelopoiesis (68). If similar processes occur in psoriasis, they could help maintain a chronically primed state for inflammatory reactivation by continuously supplying primed myeloid cells to the circulation.

From the perspective of the systemic immune set-point model, trained innate immunity provides a plausible mechanism by which inflammatory tone remains elevated even outside periods of overt disease activity. It may help explain why the system remains unusually easy to reactivate. At present, however, the link between trained innate immune states and clinical relapse in psoriasis remains more conceptual than proven. Longitudinal studies examining whether these changes persist during remission, and whether they predict subsequent flare, are still needed.

### 4.3 Stromal inflammatory niches

A third layer of inflammatory memory may reside within the tissue itself. In psoriasis, this has become increasingly apparent from epigenomic studies showing that keratinocytes in clinically resolved lesions are not fully equivalent to those in healthy skin. Profiling approaches such as chromatin accessibility mapping and single-cell epigenomics have identified persistent epigenetic differences in resolved psoriatic epidermis (69–72). Many of these changes occur in regulatory regions linked to inflammatory and stress-response genes, suggesting that keratinocytes remain poised for rapid reactivation. Emerging evidence further suggests that metabolic–epigenetic coupling within keratinocytes, including post-translational modifications such as lysine succinylation, may contribute to the stabilization of this primed state, adding an additional layer to tissue-resident inflammatory memory (73).

This tissue-level persistence has often been described as molecular scarring, whereby prior inflammatory episodes leave stable molecular imprints in resident cells even after visible lesions have cleared (74). The idea is closely related to the concept of a residual disease genomic profile, in which transcriptional and epigenetic features of psoriatic inflammation continue to be detectable despite clinical resolution (70). Viewed together, these observations suggest that resolved skin is not simply healed skin. More specifically, it may represent a remodeled tissue state that remains poised for renewed inflammation.

Even so, important questions remain unresolved. Although durable epigenetic reprogramming has been described in both immune and structural compartments, its precise role in relapse is still unclear. It is not yet known whether the signatures detected in resolved lesions are merely passive remnants of prior inflammation or whether they actively facilitate reactivation. Nor is it fully understood how these stromal memory states interact with memory T cells and trained innate immune responses over time.

Conceptually, psoriasis relapse reflects the temporal persistence of inflammatory memory across both immune and tissue compartments. Memory T cells preserve antigen-experienced inflammatory potential, trained innate immunity sustains systemic responsiveness, and stromal inflammatory niches create local tissue conditions that favor immune-cell recruitment and signal amplification. These layers are unlikely to act in isolation. Instead, their interaction may stabilize an elevated systemic immune set-point and render the disease prone to recurrence. In this sense, relapse does not arise from a single residual reservoir, but from the coordinated persistence of inflammatory memory across skin, blood and immune tissue. The same persistent inflammatory readiness that supports recurrence over time may also contribute to how psoriasis extends across organs in spatial terms.

## 5 Spatial consequences: systemic manifestations across organs

**Figure 3:**
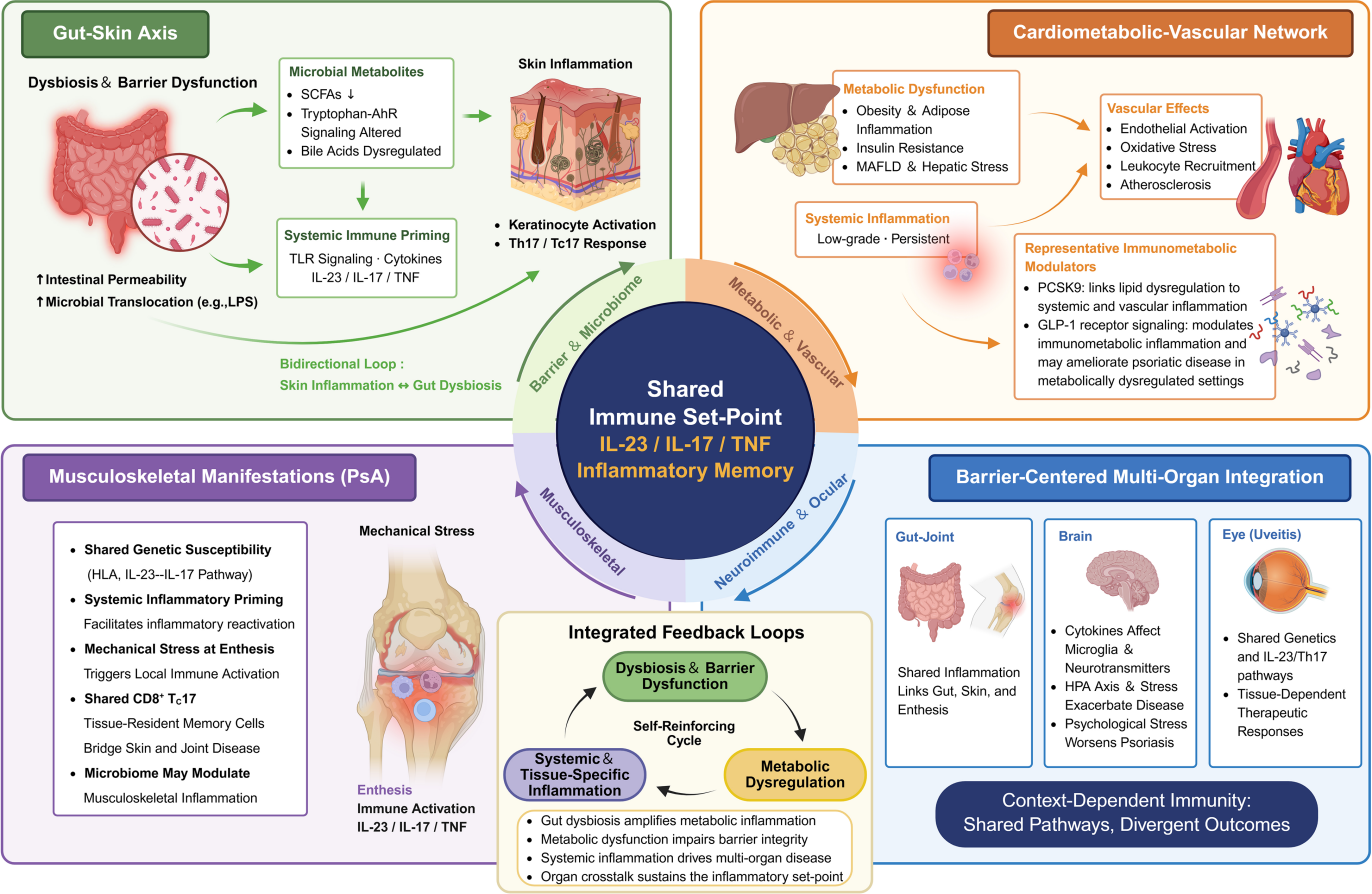
Spatial consequences of systemic immune set-point dysregulation: organ-specific translation of a shared inflammatory program in psoriasis. This image illustrates how a shared inflammatory set-point is translated into distinct but interconnected extra-cutaneous disease domains, including cardiometabolic-vascular, musculoskeletal, barrier-associated, ocular, and neuroimmune manifestations. These organ-specific outcomes are shaped by tissue context and sustained by integrated feedback among barrier dysfunction, metabolic disturbance, and systemic inflammation. AhR, aryl hydrocarbon receptor; GLP-1RAs, glucagon-like peptide-1 receptor agonists; HLA, human leukocyte antigen; IL, interleukin; LPS, lipopolysaccharide; MASLD, metabolic dysfunction-associated steatotic liver disease; NF-κB, nuclear factor kappa B; PCSK9, proprotein convertase subtilisin/kexin type 9; PsA, psoriatic arthritis; SCFAs, short-chain fatty acids; Tc, cytotoxic T cell; TLR, Toll-like receptor; TNF, tumor necrosis factor. Created with BioRender.com.

If inflammatory memory helps explain why psoriasis persists over time, an equally important question is how the same dysregulated immune state becomes expressed across space. In this view, psoriasis-associated comorbidities can be viewed as distributed organ-level expressions of the same underlying systemic inflammatory state. This systemic state is shaped not only by circulating immune mediators but also by a network of interacting regulatory systems, including barrier tissues, metabolic organs, and neuroimmune pathways. Among these, the gut–barrier–microbiome interface provides an important modulatory layer that continuously influences systemic immune tone through microbial metabolites, barrier integrity, and low-grade endotoxin exposure. In parallel, metabolic dysfunction, mechanical stress, and tissue-specific stromal contexts further modify how this shared inflammatory background is locally interpreted. Different organs therefore do not behave as independent inflammatory compartments, but instead translate a common immune set-point into distinct yet interconnected disease phenotypes. Major comorbidity domains and their principal inflammatory links are summarized in **Table 1** and **Figure 3**.

**Table 1:** Major comorbidity domains in psoriasis and the principal mechanisms through which systemic immune set-point dysregulation is translated into organ-specific disease.

Comorbidity domain	Principal inflammatory/pathobiologic links	Representative clinical manifestations
Cardiometabolic–vascular disease	Persistent systemic inflammation, endothelial activation, oxidative stress, leukocyte recruitment, lipid–immune crosstalk, and vascular remodeling (12, 75–77)	Vascular inflammation, adverse plaque features, atherosclerosis, myocardial infarction, and stroke (14, 78–80)
Metabolic dysfunction	Adipose–liver inflammatory coupling, immunometabolic reprogramming, insulin resistance, inflammasome activation, and microbiome-related metabolic disturbance (44, 77, 81–92)	Obesity, dyslipidemia, insulin resistance, type 2 diabetes, and metabolic dysfunction-associated steatotic liver disease (93–97)
Psoriatic arthritis	Shared IL-23–IL-17/TNF-centered inflammation, stromal and myeloid activation, osteoclastogenic signaling, and mechanosensitive immune activation at the enthesis (98–103)	Peripheral arthritis, enthesitis, dactylitis, axial disease, and structural joint damage (98, 104–107)
Barrier-associated and immune-privileged inflammation	Barrier dysfunction, microbiome dysbiosis, altered microbial metabolites, mucosal immune activation, and tissue-specific cytokine effects (108–113)	Inflammatory bowel disease, uveitis, and related extra-cutaneous inflammatory manifestations (114–117)
Neuropsychiatric manifestations	Chronic cytokine exposure, neuroimmune signaling, microglial activation, altered neurotransmitter metabolism, and stress–inflammation amplification (118–120)	Depression, anxiety, and stress-associated worsening of psoriasis (111)

This table summarizes the major extra-cutaneous comorbidity domains associated with psoriasis and highlights the principal inflammatory and pathobiologic mechanisms through which systemic immune set-point dysregulation may be translated into organ-specific disease expression. Rather than representing isolated complications, these manifestations can be understood as tissue-specific outputs of a shared systemic inflammatory state.

### 5.1 Cardiometabolic–vascular network

Cardiometabolic and vascular comorbidities represent a tightly interconnected component of psoriatic disease, not as discrete clinical entities but as part of a broader immunometabolic network. Epidemiologic and genetic studies consistently demonstrate strong associations between psoriasis and obesity, diabetes, dyslipidemia, and cardiovascular disease, with emerging evidence supporting a partially directional relationship in which metabolic dysfunction acts as an upstream amplifier of inflammatory susceptibility (78–80, 93–97). Vascular disease in this setting is better viewed as a downstream manifestation of a broader cardiometabolic inflammatory continuum.

Mechanistically, chronic systemic inflammation in psoriasis intersects with immunometabolic dysregulation across adipose tissue, liver, and the vasculature. Obesity-associated adipose inflammation promotes macrophage recruitment, inflammasome activation, and type 3 immune polarization (44, 81), while hepatic stress in metabolic-associated liver disease further amplifies systemic inflammatory tone through cytokine and acute-phase mediator release (121). These signals converge on the vasculature, where endothelial activation, oxidative stress, and leukocyte recruitment drive atherogenesis (12, 75, 76). Imaging studies, including ^18F-FDG PET/CT and coronary CT angiography, provide *in vivo* evidence of this integrated process, demonstrating increased vascular inflammation and adverse plaque characteristics in psoriasis cohorts (14).

Importantly, microbiome-associated metabolic regulation represents an additional layer influencing this network. Alterations in gut microbial composition and function can contribute to insulin resistance and chronic low-grade inflammation through reduced production of anti-inflammatory metabolites such as short-chain fatty acids and increased translocation of pro-inflammatory microbial products (77, 82–84). These processes reinforce systemic immune activation and link barrier dysfunction to cardiometabolic disease.

Within this integrated system, lipid–immune crosstalk plays a critical role. PCSK9, a key regulator of lipid metabolism, is elevated in psoriasis and has been implicated in macrophage activation and endothelial inflammatory signaling (85–88). Rather than acting solely as a cardiovascular mediator, PCSK9 may function as an immunometabolic node linking lipid dysregulation with systemic and vascular inflammation. In parallel, incretin-based pathways have emerged as important modulators of immunometabolic inflammation. Glucagon-like peptide-1 receptor agonists (GLP-1RAs), widely used for the treatment of type 2 diabetes and obesity, exert anti-inflammatory effects by suppressing NF-κB signaling and pro-inflammatory cytokine production while improving metabolic homeostasis (89, 90). Clinical and translational studies further suggest that GLP-1RA therapy may ameliorate psoriatic disease activity in patients with metabolic comorbidities, supporting a bidirectional link between metabolic regulation and cutaneous inflammation (91, 92). In aggregate, these observations reinforce the concept that cardiometabolic and vascular manifestations arise from a shared inflammatory network in which metabolic and microbial factors actively shape immune activation and disease expression.

### 5.2 Musculoskeletal manifestations: PsA as a mechanosensitive manifestation

Psoriatic arthritis (PsA) is the most common musculoskeletal comorbidity of psoriasis, affecting approximately 20–30% of patients, with an incidence of 1–3 cases per 100 patient-years (98). Genome-wide association studies have identified shared genetic susceptibility between psoriasis and PsA, including HLA-C*06:02, HLA-B alleles, and variants in cytokine signaling pathways related to the IL-23–IL-17 axis (104, 105). Consistent with this overlap, Mendelian randomization analyses suggest that genetic liability to psoriasis contributes to susceptibility to PsA, although the magnitude of causal estimates varies across datasets (106, 107).

Psoriasis and PsA share IL-23–IL-17-centered immune activation along with broader TNF-associated and Th1-related inflammatory pathways across the skin, joints, entheses, digits, tendons, and axial skeleton (98). This pathway coordinates lymphocyte-mediated activation of joint-resident stromal and myeloid cells and integrates inflammatory and bone-resorptive programs that drive arthritic damage (99). Mechanical stress at the enthesis can further activate IL-23-responsive resident T cells, linking biomechanical signals to immune activation (100). Shared CD8+ T cell clones, characterized by a type 17 tissue-resident memory phenotype, are thought to bridge skin and joint inflammation (101).

Emerging evidence also suggests that microbiome-associated immune modulation may contribute to musculoskeletal inflammation, although this field remains at an early stage (102, 103). Within the systemic immune set-point framework proposed in this review, these observations support a model in which a persistently primed systemic immune state allows tissue-specific triggers—particularly mechanical stress—to translate a shared inflammatory background into sustained joint disease. PsA thus emerges as a mechanosensitive manifestation of broader systemic immune dysregulation.

### 5.3 Barrier-centered multi-organ integration in psoriasis.

Psoriasis is linked to a broader network of immune-mediated manifestations involving barrier tissues, immune-privileged sites, musculoskeletal structures, and neuroimmune systems, which function as interconnected nodes within a systemic inflammatory continuum. Within this network, the gut remains an important disease-relevant organ in its own right, while also increasingly being viewed as a potential upstream modulator of systemic inflammatory tone (108, 109). Through barrier dysfunction, microbiome dysbiosis, altered microbial metabolites, and mucosal immune signaling, the gut may influence not only cutaneous inflammation but also extra-cutaneous disease expression. This concept has been increasingly framed in psoriasis as a gut–brain–skin axis, in which intestinal, neural, and cutaneous immune pathways are functionally linked (114, 115), related barrier-centered models have also been proposed in psoriatic arthritis, further supporting the idea that gut-derived perturbations may propagate inflammatory signals across skin, joints, and neuroimmune circuits (110). Within this broader framework, musculoskeletal, and neuropsychiatric manifestations can be understood as tissue-specific outputs of a shared systemic inflammatory state, shaped by both local tissue context and inter-organ immune crosstalk. Notably, this interaction is not unidirectional. Systemic inflammatory mediators associated with psoriasis can in turn alter intestinal homeostasis and microbial composition, establishing a dynamic and self-reinforcing feedback loop between barrier dysfunction and systemic inflammation (111). This bidirectional coupling provides a mechanistic basis for the coexistence and mutual amplification of cutaneous and intestinal disease.

This framework can be extended into a broader barrier–ocular axis. Ocular involvement, particularly uveitis, illustrates how immune-privileged tissues participate in this shared inflammatory network (116, 117). Uveitis shares partial genetic susceptibility and convergent inflammatory pathways with psoriasis, particularly along the IL-23/Th17 axis, reinforcing the concept of a common immunological framework (112, 113).

The neuropsychiatric dimension of psoriasis further expands this network. Depression represents a key example of the interaction between chronic inflammation and neuroimmune regulation (118). In addition to psychosocial burden, systemic inflammatory mediators—including TNF, IL-6, and IL-17—can influence central nervous system function through microglial activation, neuroinflammation, and altered neurotransmitter metabolism (119). Dysregulation of the hypothalamic–pituitary–adrenal axis may further amplify this process, linking chronic inflammation with stress-mediated neuroendocrine signaling (111). Conversely, psychological stress can exacerbate cutaneous inflammation through neuroimmune pathways, including mechanisms such as the prolactin–NR4A1–midkine axis in dermal fibroblasts (120).

Crucially, shared immunological pathways do not translate into uniform therapeutic responses across tissues. Despite a common IL-23/Th17-centered inflammatory background, targeted interventions exhibit context-dependent effects. For example, IL-17 plays a pro-inflammatory role in psoriasis but may exert protective functions in maintaining intestinal barrier integrity, consistent with the limited efficacy or potential exacerbation of inflammatory bowel disease observed with IL-17 blockade (122, 123). Similarly, therapeutic responses in ocular inflammation remain variable despite strong mechanistic rationale (124, 125). IL-23–targeted therapies show efficacy in cutaneous and intestinal disease but demonstrate inconsistent outcomes in ocular involvement (126–128).

Beyond the well-established comorbidity domains, epidemiologic studies have also reported an association between psoriasis and malignancy, particularly non-melanoma skin cancer and lymphoproliferative disorders (129, 130). This association appears modest and is likely influenced by multiple factors, including cumulative inflammatory burden, immunosuppressive therapies, phototherapy exposure, and shared lifestyle risks. Within the immune set-point framework, chronic systemic inflammation and altered immune surveillance may contribute to tumorigenesis. At the same time, key inflammatory pathways implicated in psoriasis, including IL-17 and TNF signaling, exhibit context-dependent effects in cancer biology, with both pro- and anti-tumor activities reported (131–135). These observations indicate that malignancy represents a heterogeneous and context-dependent systemic outcome shaped by immune dysregulation cooperate with environmental and therapeutic influences.

In psoriasis, a shared systemic inflammatory milieu is expressed differently across tissues, where local environmental constraints, barrier integrity, and regulatory circuits shape how disease becomes manifest. This tissue-dependent filtering helps explain why comorbidities emerge as distinct yet interconnected features spanning vascular, metabolic, musculoskeletal, neuroimmune, and barrier systems.

## 6 Integrating temporal persistence and spatial disease expression

Disease relapse and psoriasis-associated comorbidities arise as parallel consequences of a shared dysregulated immune state. Both unfold within the setting of a persistently remodeled systemic immune set-point, in which cumulative inflammatory burden, reflected in circulating inflammatory signals, immune-cell reprogramming, and multi-organ inflammatory engagement, shapes disease trajectory across time and tissues. As this inflammatory burden persists, it not only sustains inflammatory memory and leaves the system in a state of heightened inflammatory readiness, but may also extend pathogenic activity beyond the skin to involve additional organs, contributing to cardiometabolic disease, psoriatic arthritis, and other systemic manifestations. Inter-individual variation in these outcomes may reflect differences in both systemic inflammatory priming and tissue-specific susceptibility.

The temporal and spatial dimensions of psoriasis are thus intrinsically linked. In many patients, extra-cutaneous manifestations emerge progressively, with skin disease preceding broader systemic involvement, as conceptualized in the psoriasis march (136). Conversely, prolonged disease duration and sustained inflammatory exposure are associated with increased vulnerability to both relapse and multi-organ comorbidity (13, 137). Immune memory may be one important contributor to this transition, particularly in the progression from cutaneous psoriasis to psoriatic arthritis (101, 138). Supporting this view, recent work has identified distinct circulating immune endotypes—including memory CD4+ T cell, nonclassical T cell, and terminal effector/Th1 profiles—that stratify PsA phenotypes according to inflammatory severity and therapeutic response, further implicating systemic immune activation and memory in disease progression (139). In this context, spatial expansion of inflammation and temporal persistence of immune memory can be viewed as coordinated dimensions of a unified pathogenic process.

In sum, these observations support an integrated model of psoriasis in which recurrent skin disease represents one component of a broader systemic inflammatory circuit. Within this circuit, the systemic immune milieu helps shape both where disease manifests and how persistently it recurs. Therapeutically, this perspective suggests that effective long-term management should extend beyond skin clearance to include sustained reduction of systemic inflammatory burden, progressive normalization of the dysregulated immune set-point, and restoration of immune homeostasis.

## 7 Clinical implications of systemic immune set-point dysregulation

The concept of systemic immune set-point dysregulation also carries practical implications for clinical care. When psoriasis is viewed as a disorder of persistent immune priming and maladaptive set-point remodeling, several aspects of management come into sharper focus. These include how disease severity is assessed, how patients are biologically stratified, when systemic therapy is introduced, how comorbidities are monitored, and how relapse may be more effectively prevented.

### 7.1 Rethinking disease assessment: beyond cutaneous severity

Disease severity in psoriasis is still assessed largely through cutaneous measures such as the Psoriasis Area and Severity Index (PASI) and body surface area (BSA). These remain essential in routine practice, particularly for evaluating visible disease burden and treatment response. At the same time, they reflect only one part of a broader systemic immune disorder. A more complete assessment strategy may need to incorporate indicators of systemic inflammatory activity, including circulating inflammatory biomarkers, peripheral immune-cell phenotypes, cumulative disease duration, and comorbidity risk. As immunologic profiling becomes more accessible, disease monitoring may move closer to the underlying immune state rather than relying on skin severity alone.

### 7.2 Precision diagnosis and molecular stratification

This perspective also highlights the biological heterogeneity of psoriasis. Beneath a broadly similar clinical appearance, psoriasis likely encompasses multiple immunologic and tissue-level states. High-dimensional approaches, including single-cell transcriptomics, proteomics, immune repertoire sequencing, and spatial transcriptomics, are beginning to resolve this heterogeneity with much greater precision (140–142). At the same time, deep-learning–based computational pathology is expanding the ability to extract quantitative morphologic features from whole-slide images, offering an additional layer of phenotypic stratification that may complement molecular profiling (143). Emerging multimodal frameworks that align histologic architecture with spatial transcriptomic data further point toward biologically grounded tissue annotation and, in some settings, the inference of spatial gene-expression patterns from digital histology, although these approaches remain an emerging direction rather than an established diagnostic standard in psoriasis (144, 145). Over time, such strategies may help define biologically distinct disease endotypes more precisely, support therapeutic selection, improve prediction of treatment response, and identify patients who are more likely to follow particular comorbidity or relapse trajectories.

### 7.3 Early intervention and reconsideration of treatment timing

The systemic immune set-point model also has implications for when treatment should begin. In routine practice, management often follows a stepwise escalation strategy, with systemic agents introduced only after progression or inadequate response to topical therapy. Prolonged inflammatory exposure, however, may gradually reinforce pathogenic circuits through expansion of memory T-cell populations, trained innate immune states, and durable epigenetic imprinting. Earlier and more effective suppression of systemic inflammation may therefore help prevent these maladaptive immune configurations from becoming entrenched. Direct clinical evidence remains limited, but growing interest in treat-to-target approaches and earlier use of biologic therapy reflects increasing recognition that treatment timing may influence long-term disease course (72, 136).

### 7.4 Management of psoriasis comorbidities

A systemic view of psoriasis also changes how comorbidities are approached in clinical care. They are not peripheral to the disease process. Instead, they form part of its broader clinical expression and should be managed accordingly. Care models that consider dermatologic activity, systemic inflammatory burden, and comorbidity risk together are likely to be more effective over the long term. In practical terms, this includes routine screening for cardiometabolic risk factors, early recognition of musculoskeletal symptoms suggestive of psoriatic arthritis, and close coordination among dermatologists, rheumatologists, and primary care physicians.

### 7.5 Maintenance therapy and prevention of relapse

Although targeted cytokine blockade can induce rapid and sometimes near-complete skin clearance, relapse after treatment withdrawal remains common. This suggests that clinical remission does not necessarily indicate normalization of the underlying immune state. From a therapeutic perspective, two broad directions emerge. One is to achieve deeper suppression of partially overlapping inflammatory pathways. The other is to intervene at upstream mechanisms that sustain systemic inflammatory priming.

Dual inhibition of IL-17A and IL-17F, exemplified by bimekizumab, illustrates how more complete targeting of related cytokine pathways can produce high levels of skin clearance (137–139). Newer biologic formats and small-molecule agents, including the TYK2 inhibitor deucravacitinib, further point to the potential for sustained modulation of intracellular inflammatory signaling (146–148). Looking further ahead, upstream immune-priming mechanisms may become an additional therapeutic focus. Kinase-based strategies such as Bruton’s tyrosine kinase (BTK) inhibition may suppress psoriasis-like inflammation by modulating innate and B-cell-driven signaling pathways that contribute to systemic inflammatory readiness (149). Taken together, these developments suggest a gradual shift in therapeutic thinking, with greater emphasis on durable control of systemic inflammation and less reliance on episodic suppression of visible flares.

## 8 Conclusion

This review supports the view that psoriasis is a systemic disorder shaped by immune set-point dysregulation. Within this framework, persistently elevated inflammatory burden may help explain both the development of multi-organ comorbidities and the tendency for relapse despite effective control of cutaneous lesions. Recognizing psoriasis as a systemic immune disorder has important implications for disease assessment, comorbidity risk, and long-term treatment strategy. Beyond skin clearance alone, the broader goal is to achieve durable suppression of systemic inflammation and move psoriasis care closer to disease modification.

## References

[1] Armstrong AW Blauvelt A Callis Duffin K Huang Y-H Savage LJ Guo L . Psoriasis. Nat Rev Dis Primers. (2025) 11:45. doi:10.1038/s41572-025-00630-5. PMID: 40571687

[2] Svedbom A Mallbris L González-Cantero Á Playford M Wu C Mehta NN . Skin inflammation, systemic inflammation, and cardiovascular disease in psoriasis. JAMA Dermatol. (2025) 161:81–6. doi:10.1001/jamadermatol.2024.4433. PMID: 39565616 PMC11579891

[3] Raimondo MG Mohammadian H Angeli MR Alivernini S Fedorchenko V Huang K . Skin-derived myeloid precursors and joint-resident fibroblasts spread psoriatic disease from skin to joints. Nat Immunol. (2026) 27:35–47. doi:10.1038/s41590-025-02351-z. PMID: 41482544 PMC12764428

[4] R P Iyk I E K M A Cem G Dm A . National, regional, and worldwide epidemiology of psoriasis: systematic analysis and modelling study. BMJ (Clinical Res Ed). (2020) 369:m1590. doi:10.1136/bmj.m1590. PMID: 32467098 PMC7254147

[5] Wei J Wang Y Chen Y Wang Z Dai X Gelfand JM . Global burden of psoriasis from 1990 to 2021 and potential factors: a systematic analysis. J Invest Dermatol. (2026) 146:1034–1045.e22. doi:10.1016/j.jid.2025.08.038. PMID: 40930460

[6] Kwong AC Ordovas-Montanes J . Deconstructing inflammatory memory across tissue set points using cell circuit motifs. J Allergy Clin Immunol. (2024) 154:1095–105. doi:10.1016/j.jaci.2024.09.014. PMID: 39341577

[7] Francis L Capon F Smith CH Haniffa M Mahil SK . Inflammatory memory in psoriasis: from remission to recurrence. J Allergy Clin Immunol. (2024) 154:42–50. doi:10.1016/j.jaci.2024.05.008. PMID: 38761994

[8] Dong C Sun L Wang Y Lin J-M Lu X Chen J . CXCR6 sustains TRM-driven psoriasis relapse by CXCL16 chemotaxis and curcumol targeting. J Adv Res. (2025):S2090-1232(25)00861–6. doi:10.1016/j.jare.2025.10.066. PMID: 41177431

[9] Hong D Xiong H Lu S Ma J Shi Z . Metabolic regulation of the immune cell in psoriasis: mechanisms and interventions. Curr Opin Immunol. (2025) 96:102614. doi:10.1016/j.coi.2025.102614. PMID: 40674835

[10] Chen J Liu J Cao X . Functional and metabolic heterogeneity of dendritic cells in self-tolerance and autoimmunity. Immunol Rev. (2025) 336:e70068. doi:10.1111/imr.70068. PMID: 41219689

[11] Kao Y-S Lauterbach M Lopez Krol A Distler U Godoy GJ Klein M . Metabolic reprogramming of interleukin-17-producing γδ T cells promotes ACC1-mediated de novo lipogenesis under psoriatic conditions. Nat Metab. (2025) 7:966–84. doi:10.1038/s42255-025-01276-z. PMID: 40360755 PMC12116387

[12] Gelfand JM Song WB Langan SM Garshick MS . Cardiodermatology: the heart of the connection between the skin and cardiovascular disease. Nat Rev Cardiol. (2025) 22:354–71. doi:10.1038/s41569-024-01097-9. PMID: 39537837

[13] Weber B Merola JF Husni ME Di Carli M Berger JS Garshick MS . Psoriasis and cardiovascular disease: novel mechanisms and evolving therapeutics. Curr Atheroscler Rep. (2021) 23:67. doi:10.1007/s11883-021-00963-y. PMID: 34468875 PMC9744099

[14] Patel NH Osborne MT Teague H Parel P Svirydava M Sorokin AV . Heightened splenic and bone marrow uptake of 18F-FDG PET/CT is associated with systemic inflammation and subclinical atherosclerosis by CCTA in psoriasis: an observational study. Atherosclerosis. (2021) 339:20–6. doi:10.1016/j.atherosclerosis.2021.11.008. PMID: 34808541 PMC8723715

[15] Kaiser H Kvist-Hansen A Krakauer M Gørtz PM Henningsen KMA Wang X . Association between vascular inflammation and inflammation in adipose tissue, spleen, and bone marrow in patients with psoriasis. Life (Basel). (2021) 11:305. doi:10.3390/life11040305. PMID: 33915972 PMC8065955

[16] Liu S He M Jiang J Duan X Chai B Zhang J . Triggers for the onset and recurrence of psoriasis: a review and update. Cell Commun Signal. (2024) 22:108. doi:10.1186/s12964-023-01381-0. PMID: 38347543 PMC10860266

[17] Chen L Cheng L . New hope for the treatment of recurrent and refractory psoriasis: NK cell immunotherapy-a scientometric analysis. Front Immunol. (2025) 16:1656398. doi:10.3389/fimmu.2025.1656398. PMID: 41209017 PMC12590505

[18] Prajapati VH Lynde CW Gooderham MJ Hong H-H Kirchhof MG Lansang P . Considerations for defining and diagnosing generalized pustular psoriasis. J Eur Acad Dermatol Venereol. (2025) 39:487–97. doi:10.1111/jdv.20310. PMID: 39239977 PMC11851258

[19] Murphy KM Weaver C . Janeway’s immunobiology. New York, NY: W. W. Norton & Company (2022).

[20] Goodnow CC Sprent J Fazekas de St Groth B Vinuesa CG . Cellular and genetic mechanisms of self tolerance and autoimmunity. Nature. (2005) 435:590–7. doi:10.1038/nature03724. PMID: 15931211

[21] Hogquist KA Baldwin TA Jameson SC . Central tolerance: learning self-control in the thymus. Nat Rev Immunol. (2005) 5:772–82. doi:10.1038/nri1707. PMID: 16200080

[22] Dand N Stuart PE Bowes J Ellinghaus D Nititham J Saklatvala JR . GWAS meta-analysis of psoriasis identifies new susceptibility alleles impacting disease mechanisms and therapeutic targets. Nat Commun. (2025) 16:2051. doi:10.1038/s41467-025-56719-8. PMID: 40021644 PMC11871359

[23] Chen J Yin H Li S Li C . Autoimmune skin disease pathogenesis: a chronological immune cascade and multi-hit model. Immun Inflammation. (2026) 2:4. doi:10.1007/s44466-025-00017-x. PMID: 30311153

[24] Yu W-W Barrett JNP Tong J Lin M-J Marohn M Devlin JC . Skin immune-mesenchymal interplay within tertiary lymphoid structures promotes autoimmune pathogenesis in hidradenitis suppurativa. Immunity. (2024) 57:2827–2842.e5. doi:10.1016/j.immuni.2024.11.010. PMID: 39662091 PMC12404358

[25] Chen J Bai Y Xue K Li Z Zhu Z Li Q . CREB1-driven CXCR4hi neutrophils promote skin inflammation in mouse models and human patients. Nat Commun. (2023) 14:5894. doi:10.1038/s41467-023-41484-3. PMID: 37736772 PMC10516899

[26] Xia Y Lan J Yang J Yuan S Xie X Du Q . Saturated fatty acid-induced neutrophil extracellular traps contribute to exacerbation and biologic therapy resistance in obesity-related psoriasis. Cell Mol Immunol. (2025) 22:597–611. doi:10.1038/s41423-025-01278-7. PMID: 40169704 PMC12125246

[27] Nazimek K Bryniarski K . Macrophage functions in psoriasis: lessons from mouse models. Int J Mol Sci. (2024) 25:5306. doi:10.3390/ijms25105306. PMID: 38791342 PMC11121292

[28] Sieminska I Pieniawska M Grzywa TM . The immunology of psoriasis-current concepts in pathogenesis. Clin Rev Allergy Immunol. (2024) 66:164–91. doi:10.1007/s12016-024-08991-7. PMID: 38642273 PMC11193704

[29] Zhang B Mei J Liao Q Zhou S Huang H Liu H . Multitranscriptome analysis reveals stromal cells in the papillary dermis to promote angiogenesis in psoriasis vulgaris. Br J Dermatol. (2025) 192:672–83. doi:10.1093/bjd/ljae459. PMID: 39569441

[30] Zhou X Chen Y Cui L Shi Y Guo C . Advances in the pathogenesis of psoriasis: from keratinocyte perspective. Cell Death Dis. (2022) 13:81. doi:10.1038/s41419-022-04523-3. PMID: 35075118 PMC8786887

[31] Ss P D S . Systemic psoriasis: from molecular mechanisms to global management strategies. Clin Rev Allergy Immunol. (2025) 68(1):79. doi:10.1007/s12016-025-09089-4. PMID: 40775488

[32] Dand N Mahil SK Capon F Smith CH Simpson MA Barker JN . Psoriasis and genetics. Acta Derm Venereol. (2020) 100:adv00030. doi:10.2340/00015555-3384. PMID: 31971603 PMC9128944

[33] Swindell WR Sarkar MK Liang Y Xing X Gudjonsson JE . Cross-disease transcriptomics: unique IL-17A signaling in psoriasis lesions and an autoimmune PBMC signature. J Invest Dermatol. (2016) 136:1820–30. doi:10.1016/j.jid.2016.04.035. PMID: 27206706 PMC5234565

[34] Damara A Wegner J Trzeciak ER Kolb A Nastaranpour M Khatri R . LL37/self-DNA complexes mediate monocyte reprogramming. Clin Immunol. (2024) 265:110287. doi:10.1016/j.clim.2024.110287. PMID: 38909973

[35] Wu D Hailer AA Wang S Yuan M Chan J El Kurdi A . A single-cell atlas of IL-23 inhibition in cutaneous psoriasis distinguishes clinical response. Sci Immunol. (2024) 9:eadi2848. doi:10.1126/sciimmunol.adi2848. PMID: 38277466

[36] Francis L McCluskey D Ganier C Jiang T Du-Harpur X Gabriel J . Single-cell analysis of psoriasis resolution demonstrates an inflammatory fibroblast state targeted by IL-23 blockade. Nat Commun. (2024) 15:913. doi:10.1038/s41467-024-44994-w. PMID: 38291032 PMC10828502

[37] Kim J Lee J Lee J Kim K Li X Zhou W . Psoriasis harbors multiple pathogenic type 17 T-cell subsets: selective modulation by risankizumab. J Allergy Clin Immunol. (2025) 155:1898–912. doi:10.1016/j.jaci.2025.02.008. PMID: 39978685 PMC12145251

[38] Zhang B Roesner LM Traidl S Koeken VACM Xu C-J Werfel T . Single-cell profiles reveal distinctive immune response in atopic dermatitis in contrast to psoriasis. Allergy. (2023) 78:439–53. doi:10.1111/all.15486. PMID: 35986602

[39] Phadungsaksawasdi P Fujiyama T Kurihara K Ito T Honda T Tokura Y . PD-1 expression defines epidermal CD8+CD103+ T cells preferentially producing IL-17A and using skewed TCR repertoire in psoriasis. J Invest Dermatol. (2021) 141:2426–2435.e5. doi:10.1016/j.jid.2021.03.011. PMID: 33845077

[40] Povoleri GAM Durham LE Gray EH Lalnunhlimi S Kannambath S Pitcher MJ . Psoriatic and rheumatoid arthritis joints differ in the composition of CD8+ tissue-resident memory T cell subsets. Cell Rep. (2023) 42:112514. doi:10.1016/j.celrep.2023.112514. PMID: 37195862 PMC10790246

[41] Ma F Plazyo O Billi AC Tsoi LC Xing X Wasikowski R . Single cell and spatial sequencing define processes by which keratinocytes and fibroblasts amplify inflammatory responses in psoriasis. Nat Commun. (2023) 14:3455. doi:10.1038/s41467-023-39020-4. PMID: 37308489 PMC10261041

[42] Ma J Chen J Xue K Yu C Dang E Qiao H . LCN2 mediates skin inflammation in psoriasis through the SREBP2–NLRC4 axis. J Invest Dermatol. (2022) 142:2194–2204.e11. doi:10.1016/j.jid.2022.01.012. PMID: 35120997

[43] Li Q Pang B Dang E Wang G . Endothelial dysfunction in psoriasis: an integrative review. J Invest Dermatol. (2024) 144:1935–42. doi:10.1016/j.jid.2024.02.013. PMID: 38493385

[44] Jang J Park M Kim HJ Jung Y . Reprogramming immunity at the metabolic-epidermal interface in obesity-associated psoriasis. Cytokine Growth Factor Rev. (2026) 88:32–46. doi:10.1016/j.cytogfr.2026.01.003. PMID: 41534195

[45] Subudhi I Konieczny P Prystupa A Castillo RL Sze-Tu E Xing Y . Metabolic coordination between skin epithelium and type 17 immunity sustains chronic skin inflammation. Immunity. (2024) 57:1665–1680.e7. doi:10.1016/j.immuni.2024.04.022. PMID: 38772365 PMC11236527

[46] Liu Y Qin G Meng Z Du T Wang X Tang Y . IL-1β, IL-17A and combined phototherapy predicts higher while previous systemic biologic treatment predicts lower treatment response to etanercept in psoriasis patients. Inflammopharmacology. (2019) 27:57–66. doi:10.1007/s10787-018-0530-9. PMID: 30242748

[47] Piaserico S Orlando G Messina F . Psoriasis and cardiometabolic diseases: shared genetic and molecular pathways. Int J Mol Sci. (2022) 23:9063. doi:10.3390/ijms23169063. PMID: 36012327 PMC9409274

[48] Ramessur R Corbett M Marshall D Acencio ML Barbosa IA Dand N . Biomarkers of disease progression in people with psoriasis: a scoping review. Br J Dermatol. (2022) 187:481–93. doi:10.1111/bjd.21627. PMID: 35482474 PMC9796834

[49] Elnabawi YA Garshick MS Tawil M Barrett TJ Fisher EA Lo Sicco K . CCL20 in psoriasis: a potential biomarker of disease severity, inflammation, and impaired vascular health. J Am Acad Dermatol. (2021) 84:913–20. doi:10.1016/j.jaad.2020.10.094. PMID: 33259876 PMC8049184

[50] Beygi S Lajevardi V Abedini R . C-reactive protein in psoriasis: a review of the literature. J Eur Acad Dermatol Venereol. (2014) 28:700–11. doi:10.1111/jdv.12257. PMID: 23998353

[51] González-Cantero A Ortega-Quijano D Álvarez-Díaz N Ballester MA Jimenez-Gomez N Jaen P . Impact of biological agents on imaging and biomarkers of cardiovascular disease in patients with psoriasis: a systematic review and meta-analysis of randomized placebo-controlled trials. J Invest Dermatol. (2021) 141:2402–11. doi:10.1016/j.jid.2021.03.024. PMID: 33891953

[52] Ma J Sun X Wu Z Qi R Niu J . Correlation between the systemic immune inflammation index and risk of psoriasis: results from NHANES. Eur J Dermatol. (2024) 34:31–9. doi:10.1684/ejd.2024.4610. PMID: 38557456

[53] Näslund-Koch C Kvist-Hansen A Bojesen SE Skov L Kobylecki CJ Vedel-Krogh S . Low-grade systemic inflammation is associated with risk of psoriasis in a general population study of more than 100–000 individuals. Br J Dermatol. (2025) 193:250–8. doi:10.1093/bjd/ljaf147. PMID: 40249082

[54] Joshi AA Lerman JB Aberra TM Afshar M Teague HL Rodante JA . GlycA is a novel biomarker of inflammation and subclinical cardiovascular disease in psoriasis. Circ Res. (2016) 119:1242–53. doi:10.1161/CIRCRESAHA.116.309637. PMID: 27654120 PMC5215065

[55] Choe YB Hwang YJ Hahn HJ Jung JW Jung HJ Lee YW . A comparison of serum inflammatory cytokines according to phenotype in patients with psoriasis. Br J Dermatol. (2012) 167:762–7. doi:10.1111/j.1365-2133.2012.11038.x. PMID: 22564054

[56] Masson Regnault M Shourick J Jendoubi F Tauber M Paul C . Time to relapse after discontinuing systemic treatment for psoriasis: a systematic review. Am J Clin Dermatol. (2022) 23:433–47. doi:10.1007/s40257-022-00679-y. PMID: 35489008 PMC9055370

[57] Netea MG Schlitzer A Placek K Joosten LAB Schultze JL . Innate and adaptive immune memory: an evolutionary continuum in the host’s response to pathogens. Cell Host Microbe. (2019) 25:13–26. doi:10.1016/j.chom.2018.12.006. PMID: 30629914

[58] Masopust D Awasthi A Bosselut R Brooks DG Buggert M Chamoto K . Guidelines for T cell nomenclature. Nat Rev Immunol. (2025) 26(4):298–313. doi:10.1038/s41577-025-01238-2. PMID: 41254224

[59] Liu J Chang H-W Huang Z-M Nakamura M Sekhon S Ahn R . Single-cell RNA sequencing of psoriatic skin identifies pathogenic Tc17 cell subsets and reveals distinctions between CD8+ T cells in autoimmunity and cancer. J Allergy Clin Immunol. (2021) 147:2370–80. doi:10.1016/j.jaci.2020.11.028. PMID: 33309739 PMC9179181

[60] Zhuang W Zhang Q Kong Q Hui Y Shen J Zhang C . Integrated single-cell and spatial transcriptomic analysis reveals a pathological niche formed by FAP+ fibroblasts, immune, and endothelial cells in psoriatic lesions. Clin Cosmet Investig Dermatol. (2025) 18:2323–40. doi:10.2147/CCID.S541106. PMID: 40963886 PMC12439697

[61] Migayron L Merhi R Seneschal J Boniface K . Resident memory T cells in nonlesional skin and healed lesions of patients with chronic inflammatory diseases: appearances can be deceptive. J Allergy Clin Immunol. (2024) 153:606–14. doi:10.1016/j.jaci.2023.11.017. PMID: 37995858

[62] Buggert M Price DA Mackay LK Betts MR . Human circulating and tissue-resident memory CD8+ T cells. Nat Immunol. (2023) 24:1076–86. doi:10.1038/s41590-023-01538-6. PMID: 37349380

[63] den Braanker H Razawy W Wervers K Mus A-M Davelaar N Kok MR . Characterizing memory T helper cells in patients with psoriasis, subclinical, or early psoriatic arthritis using a machine learning algorithm. Arthritis Res Ther. (2022) 24:28. doi:10.1186/s13075-021-02714-5. PMID: 35045868 PMC8767727

[64] Diani M Galasso M Cozzi C Sgambelluri F Altomare A Cigni C . Blood to skin recirculation of CD4+ memory T cells associates with cutaneous and systemic manifestations of psoriatic disease. Clin Immunol. (2017) 180:84–94. doi:10.1016/j.clim.2017.04.001. PMID: 28392462

[65] Ochando J Mulder WJM Madsen JC Netea MG Duivenvoorden R . Trained immunity - basic concepts and contributions to immunopathology. Nat Rev Nephrol. (2023) 19:23–37. doi:10.1038/s41581-022-00633-5. PMID: 36253509 PMC9575643

[66] Qu D Zhao L Zhang Q Li R Li Y Wang H . Reprogramming the impact of glutamine metabolism on controlling the immunoinflammatory milieu in psoriasis. Biochem Pharmacol. (2025) 242:117217. doi:10.1016/j.bcp.2025.117217. PMID: 40789373

[67] Dopytalska K Ciechanowicz P Wiszniewski K Szymańska E Walecka I . The role of epigenetic factors in psoriasis. Int J Mol Sci. (2021) 22:9294. doi:10.3390/ijms22179294. PMID: 34502197 PMC8431057

[68] Kohutek ZA Caslin HL Fehrenbach DJ Heimlich JB Brown JD Madhur MS . Bone marrow niche in cardiometabolic disease: mechanisms and therapeutic potential. Circ Res. (2025) 136:325–53. doi:10.1161/CIRCRESAHA.124.323778. PMID: 39883790 PMC11790260

[69] Zhang Z Liu L Shen Y Meng Z Chen M Lu Z . Characterization of chromatin accessibility in psoriasis. Front Med. (2022) 16:483–95. doi:10.1007/s11684-021-0872-3. PMID: 34669155

[70] Suárez-Fariñas M Fuentes-Duculan J Lowes MA Krueger JG . Resolved psoriasis lesions retain expression of a subset of disease-related genes. J Invest Dermatol. (2011) 131:391–400. doi:10.1038/jid.2010.280. PMID: 20861854 PMC3021088

[71] Furue M Kadono T . Inflammatory skin march” in atopic dermatitis and psoriasis. Inflammation Res. (2017) 66:833–42. doi:10.1007/s00011-017-1065-z. PMID: 28620798

[72] Chen J Zhou G Sha Y Xiao J Chen C Deng W . Disease modification in psoriasis through early interleukin 17 inhibitor intervention: a retrospective cohort study. J Am Acad Dermatol. (2025) 93:1000–7. doi:10.1016/j.jaad.2025.06.019. PMID: 40516878

[73] Niu X Zhao Y Zhang T Sun Y Wei Z Fu K . Comprehensive succinylome analyses reveal that hyperthermia upregulates lysine succinylation of annexin A2 by downregulating sirtuin7 in human keratinocytes. J Transl Int Med. (2024) 12:424–36. doi:10.2478/jtim-2022-0061. PMID: 39360157 PMC11444469

[74] Kasprowicz-Furmańczyk M Narbutt J Borzęcki A Owczarczyk-Saczonek A . Does molecular scarring in psoriasis exist? A review of the literature. Postepy Dermatol Alergol. (2023) 40:473–80. doi:10.5114/ada.2023.129322. PMID: 37692280 PMC10485766

[75] Schaller T Ringen J Fischer B Bieler T Perius K Knopp T . Reactive oxygen species produced by myeloid cells in psoriasis as a potential biofactor contributing to the development of vascular inflammation. Biofactors. (2023) 49:861–74. doi:10.1002/biof.1949. PMID: 37139784

[76] Ma L Yang W Gao W Liu X Dong M An G . IL-17 as a therapeutic target in cardiovascular diseases: mechanistic insights and translational opportunities. Pharmacol Res. (2025) 219:107879. doi:10.1016/j.phrs.2025.107879. PMID: 40714302

[77] Jardon KM Umanets A Gijbels A Trouwborst I Hul GB Siebelink E . Distinct gut microbiota and metabolome features of tissue-specific insulin resistance in overweight and obesity. Gut Microbes. (2025) 17:2501185. doi:10.1080/19490976.2025.2501185. PMID: 40336254 PMC12064058

[78] Snekvik I Nilsen TIL Romundstad PR Saunes M . Psoriasis and cardiovascular disease risk factors: the HUNT study, Norway. J Eur Acad Dermatol Venereol. (2018) 32:776–82. doi:10.1111/jdv.14835. PMID: 29397035

[79] Bernardini N Dattola A Gemma GPA Atzori L Artosi F Biondi G . Psoriasis severity, comorbidity burden, and biologic therapy: a multicenter observational study using the Charlson Comorbidity Index. J Dermatolog Treat. (2025) 36:2562311. doi:10.1080/09546634.2025.2562311. PMID: 40990256

[80] Ramessur R Saklatvala J Budu-Aggrey A Ostaszewski M Möbus L Greco D . Exploring the link between genetic predictors of cardiovascular disease and psoriasis. JAMA Cardiol. (2024) 9:1009–17. doi:10.1001/jamacardio.2024.2859. PMID: 39292496 PMC11411451

[81] Haberman RH Ogdie A Merola JF Scher JU Eder L . The obesity-inflammation axis in psoriatic disease: mechanisms and therapeutic strategies. Nat Rev Rheumatol. (2026) 22:151–64. doi:10.1038/s41584-025-01326-6. PMID: 41286370

[82] Kordahi MC Daniel N Gewirtz AT Chassaing B . Mucus-penetrating microbiota drive chronic low-grade intestinal inflammation and metabolic dysregulation. Gut Microbes. (2025) 17:2455790. doi:10.1080/19490976.2025.2455790. PMID: 39865067 PMC11776472

[83] He Y Shaoyong W Chen Y Li M Gan Y Sun L . The functions of gut microbiota-mediated bile acid metabolism in intestinal immunity. J Adv Res. (2026) 80:351–70. doi:10.1016/j.jare.2025.05.015. PMID: 40354934 PMC12869224

[84] Mukhopadhya I Louis P . Gut microbiota-derived short-chain fatty acids and their role in human health and disease. Nat Rev Microbiol. (2025) 23:635–51. doi:10.1038/s41579-025-01183-w. PMID: 40360779

[85] Garshick MS Baumer Y Dey AK Grattan R Ng Q Teague HL . Characterization of PCSK9 in the blood and skin of psoriasis. J Invest Dermatol. (2021) 141:308–15. doi:10.1016/j.jid.2020.05.115. PMID: 32615123 PMC8493651

[86] Luan C Chen X Zhu Y Osland JM Gerber SD Dodds M . Potentiation of psoriasis-like inflammation by PCSK9. J Invest Dermatol. (2019) 139:859–67. doi:10.1016/j.jid.2018.07.046. PMID: 30395847 PMC7546417

[87] Peng Z Lv S-J Chen H Rao H Guo Z Wan Q . Disruption of PCSK9 suppresses inflammation and attenuates abdominal aortic aneurysm formation. Arterioscler Thromb Vasc Biol. (2025) 45:e1–e14. doi:10.1161/ATVBAHA.123.320391. PMID: 39588646

[88] Huang L Li Y Cheng Z Lv Z Luo S Xia Y . PCSK9 promotes endothelial dysfunction during sepsis via the TLR4/MyD88/NF-κB and NLRP3 pathways. Inflammation. (2023) 46:115–28. doi:10.1007/s10753-022-01715-z. PMID: 35930089

[89] Paschou IA Sali E Paschou SA Psaltopoulou T Nicolaidou E Stratigos AJ . The effects of GLP-1RA on inflammatory skin diseases: a comprehensive review. J Eur Acad Dermatol Venereol. (2025) 39:2047–55. doi:10.1111/jdv.20694. PMID: 40298469 PMC12645182

[90] C A O A Ld B J M R T S I . The role of GLP1 receptor agonists and multi-agonist incretin therapies for specific obesity-related health conditions: evidence and rationale for prioritisation. Curr Obes Rep. (2026) 15(1):29. doi:10.1007/s13679-026-00707-6. PMID: 41922811

[91] LinC BraunN CoreyS ThompsonBB MaK-K ChenST . Effect of GLP-1RA on mortality and cardiovascular risk in adults with psoriasis and type 2 diabetes. J Eur Acad Dermatol Venereol. (2026). doi:10.1111/jdv.70454. PMID: 41964316

[92] Olbrich H Kridin K Zirpel H Hernandez G Sadik CD Gaffal E . Glucagon-like peptide-1 receptor agonists and reduced mortality, cardiovascular and psychiatric risks in patients with psoriasis: a large-scale cohort study. Br J Dermatol. (2026) 194:59–66. doi:10.1093/bjd/ljaf346. PMID: 40897378

[93] Bellinato F Maurelli M Geat D Girolomoni G Gisondi P . Managing the patient with psoriasis and metabolic comorbidities. Am J Clin Dermatol. (2024) 25:527–40. doi:10.1007/s40257-024-00857-0. PMID: 38748391 PMC11193697

[94] Untaaveesup S Kantagowit P Ungprasert P Kitlertbanchong N Vajiraviroj T Sutithavinkul T . The risk of metabolic dysfunction-associated steatotic liver disease in moderate-to-severe psoriasis: a systematic review and meta-analysis. J Clin Med. (2025) 14:1374. doi:10.3390/jcm14041374. PMID: 40004904 PMC11855964

[95] Karlsson T Hadizadeh F Rask-Andersen M Johansson Å Ek WE . Body mass index and the risk of rheumatic disease: linear and nonlinear Mendelian randomization analyses. Arthritis Rheumatol. (2023) 75:2027–35. doi:10.1002/art.42613. PMID: 37219954

[96] Zhao SS Bellou E Verstappen SMM Cook MJ Sergeant JC Warren RB . Association between psoriatic disease and lifestyle factors and comorbidities: cross-sectional analysis and Mendelian randomization. Rheumatol (Oxford). (2023) 62:1272–85. doi:10.1093/rheumatology/keac403. PMID: 35861400 PMC9977114

[97] Budu-Aggrey A Brumpton B Tyrrell J Watkins S Modalsli EH Celis-Morales C . Evidence of a causal relationship between body mass index and psoriasis: a Mendelian randomization study. PloS Med. (2019) 16:e1002739. doi:10.1371/journal.pmed.1002739. PMID: 30703100 PMC6354959

[98] Blauvelt A Chiricozzi A . The immunologic role of IL-17 in psoriasis and psoriatic arthritis pathogenesis. Clin Rev Allergy Immunol. (2018) 55:379–90. doi:10.1007/s12016-018-8702-3. PMID: 30109481 PMC6244934

[99] Qin P Kragsnaes MS Holm DK Horn HC Nilsson AC Kjeldsen J . Clinical significance of gut microbiota community types for long-term response to fecal microbiota transplantation in patients with psoriatic arthritis. Arthritis Rheumatol. (2026) 78:320–31. doi:10.1002/art.43359. PMID: 40814761 PMC12936901

[100] Cai Y Xie S Jia X Chen D Wu D Bao W . Integrated analysis of Mendelian randomization and Bayesian colocalization reveals bidirectional causal association between inflammatory bowel disease and psoriasis. Ann Med. (2023) 55:2281658. doi:10.1080/07853890.2023.2281658. PMID: 37988718 PMC10836255

[101] Fu Y Lee C-H Chi C-C . Association of psoriasis with inflammatory bowel disease: a systematic review and meta-analysis. JAMA Dermatol. (2018) 154:1417–23. doi:10.1001/jamadermatol.2018.3631. PMID: 30422277 PMC6583370

[102] Jairath V Acosta Felquer ML Cho RJ . IL-23 inhibition for chronic inflammatory disease. Lancet. (2024) 404:1679–92. doi:10.1016/S0140-6736(24)01750-1. PMID: 39461795

[103] Welch C Wilson D Sayer AA Witham MD Jackson TA UK Geriatric Medicine Core Dataset Extended Working Group . Development of a UK core dataset for geriatric medicine research: a position statement and results from a Delphi consensus process. BMC Geriatr. (2023) 23:168. doi:10.1186/s12877-023-03805-5. PMID: 36959622 PMC10035483

[104] Duan K Wang J Chen S Chen T Wang J Wang S . Causal associations between both psoriasis and psoriatic arthritis and multiple autoimmune diseases: a bidirectional two-sample Mendelian randomization study. Front Immunol. (2024) 15:1422626. doi:10.3389/fimmu.2024.1422626. PMID: 39119335 PMC11306030

[105] Jin JQ Elhage KG Spencer RK Davis MS Hakimi M Bhutani T . Mendelian randomization studies in psoriasis and psoriatic arthritis: a systematic review. J Invest Dermatol. (2023) 143:762–776.e3. doi:10.1016/j.jid.2022.11.014. PMID: 36822971

[106] Adamopoulos IE Baraliakos X Ritchlin C . Psoriatic arthritis: diagnosis, pathogenesis, and emerging therapies. Trends Mol Med. (2025) 31:682–3. doi:10.1016/j.molmed.2025.05.003. PMID: 40500630 PMC12393800

[107] Xynogalas I Karamanakos A Galani A Kougkas N Vassilakis KD Baraliakos X . Enthesitis in spondyloarthritis. From pathogenesis to clinical presentation and imaging: similarities and differences between PsA and axSpA. Autoimmun Rev. (2026) 25(4):104014. doi:10.1016/j.autrev.2026.104014. PMID: 41791558

[108] Thye A-K Bah Y-R Law J-F Tan L-H He Y-W Wong S-H . Gut-skin axis: Unravelling the connection between the gut microbiome and psoriasis. Biomedicines. (2022) 10:1037. doi:10.3390/biomedicines10051037. PMID: 35625774 PMC9138548

[109] Wang C-Y Lin T-Y Wang T-Y Chi C-C . Ocular comorbidities of psoriasis: a systematic review and meta-analysis of observational studies. Am J Ophthalmol. (2026) 281:181–200. doi:10.1016/j.ajo.2025.09.020. PMID: 40975155

[110] VH W G E P S E D L E T L . Barrier breakdown: insights into the skin-gut axis in psoriatic arthritis. Trends Immunol. (2026) S1471-4906(26):00036–0. doi:10.1016/j.it.2026.02.005. PMID: 41826113

[111] Tang L Bi H Lin K Chen Y Xian H Li Y . The skin-brain axis in psoriasis and depression: roles of inflammation, hormones, neuroendocrine pathways, neuropeptides, and the microbiome. Psoriasis (Auckl). (2025) 15:411–28. doi:10.2147/PTT.S535900. PMID: 40860250 PMC12372838

[112] Fotiadou C Lazaridou E . Psoriasis and uveitis: links and risks. Psoriasis (Auckl). (2019) 9:91–6. doi:10.2147/PTT.S179182. PMID: 31696050 PMC6717847

[113] Navarro-Compán V Sepriano A Capelusnik D Baraliakos X . Axial spondyloarthritis. Lancet. (2025) 405:159–72. doi:10.1016/S0140-6736(24)02263-3. PMID: 39798984

[114] Chen G Chen Z-M Fan X-Y Jin Y-L Li X Wu S-R . Gut-brain-skin axis in psoriasis: a review. Dermatol Ther (Heidelb). (2021) 11:25–38. doi:10.1007/s13555-020-00466-9. PMID: 33206326 PMC7859123

[115] G Z Y J Z R Y Y W Q X C . The brain-gut-skin axis in inflammatory and disfiguring skin diseases: mechanistic insights, clinical correlations, and therapeutic strategies. Front Immunol. (2026) 17:1737303. doi:10.3389/fimmu.2026.1737303. PMID: 41836406 PMC12982022

[116] Chi C-C Tung T-H Wang J Lin Y-S Chen Y-F Hsu T-K . Risk of uveitis among people with psoriasis: a nationwide cohort study. JAMA Ophthalmol. (2017) 135:415–22. doi:10.1001/jamaophthalmol.2017.0569. PMID: 28418500 PMC5846892

[117] Brüner M Dige A Loft AG Laurberg TB Agnholt JS Clemmensen K . Spondylitis-psoriasis-enthesitis-enterocolitis-dactylitis-uveitis-peripheral synovitis (SPEED-UP) treatment. Autoimmun Rev. (2021) 20:102731. doi:10.1016/j.autrev.2020.102731. PMID: 33326852

[118] Miller AH . Advancing an inflammatory subtype of major depression. Am J Psychiatry. (2025) 182:516–24. doi:10.1176/appi.ajp.20250289. PMID: 40329642 PMC12282100

[119] Zhang W Wang C Fu T Zhang S Tian F . CD4 + T lymphocyte and cytokine profiles in depressive disorders. BMC Psychiatry. (2025) 25:911. doi:10.1186/s12888-025-07344-8. PMID: 41034792 PMC12486925

[120] Li Z Quan H Liu W Chen J Chu M Tang X . Stress-induced activation of prolactin-NR4A1-midkine axis exacerbates skin inflammation. Adv Sci (Weinh). (2026) 13:e09679. doi:10.1002/advs.202509679. PMID: 41194398 PMC12850390

[121] F C A V G B D S D K S S . Decoding the liver-heart axis in cardiometabolic diseases. Circ Res. (2025) 136(11):1335–62. doi:10.1161/CIRCRESAHA.125.325492. PMID: 40403112 PMC7617754

[122] Fauny M Moulin D D’Amico F Netter P Petitpain N Arnone D . Paradoxical gastrointestinal effects of interleukin-17 blockers. Ann Rheum Dis. (2020) 79:1132–8. doi:10.1136/annrheumdis-2020-217927. PMID: 32719044

[123] Hueber W Sands BE Lewitzky S Vandemeulebroecke M Reinisch W Higgins PDR . Secukinumab, a human anti-IL-17A monoclonal antibody, for moderate to severe Crohn’s disease: unexpected results of a randomised, double-blind placebo-controlled trial. Gut. (2012) 61:1693–700. doi:10.1136/gutjnl-2011-301668. PMID: 22595313 PMC4902107

[124] Elman SA Perez-Chada LM Armstrong A Gottlieb AB Merola JF . Psoriatic arthritis: a comprehensive review for the dermatologist-part II: screening and management. J Am Acad Dermatol. (2025) 92:985–98. doi:10.1016/j.jaad.2024.03.059. PMID: 38857766

[125] Ramiro S Nikiphorou E Sepriano A Ortolan A Webers C Baraliakos X . ASAS-EULAR recommendations for the management of axial spondyloarthritis: 2022 update. Ann Rheum Dis. (2023) 82:19–34. doi:10.1136/ard-2022-223296. PMID: 36270658

[126] Ghoreschi K Balato A Enerbäck C Sabat R . Therapeutics targeting the IL-23 and IL-17 pathway in psoriasis. Lancet. (2021) 397:754–66. doi:10.1016/S0140-6736(21)00184-7. PMID: 33515492

[127] Vuyyuru SK Shackelton LM Hanzel J Ma C Jairath V Feagan BG . Targeting IL-23 for IBD: rationale and progress to date. Drugs. (2023) 83:873–91. doi:10.1007/s40265-023-01882-9. PMID: 37266801

[128] Pepple KL Lin P . Targeting interleukin-23 in the treatment of noninfectious uveitis. Ophthalmology. (2018) 125:1977–83. doi:10.1016/j.ophtha.2018.05.014. PMID: 30458922 PMC6538052

[129] Vaengebjerg S Skov L Egeberg A Loft ND . Prevalence, incidence, and risk of cancer in patients with psoriasis and psoriatic arthritis: a systematic review and meta-analysis. JAMA Dermatol. (2020) 156:421–9. doi:10.1001/jamadermatol.2020.0024. PMID: 32074260 PMC7042857

[130] Trafford AM Parisi R Kontopantelis E Griffiths CEM Ashcroft DM . Association of psoriasis with the risk of developing or dying of cancer: a systematic review and meta-analysis. JAMA Dermatol. (2019) 155:1390–403. doi:10.1001/jamadermatol.2019.3056. PMID: 31617868 PMC6802036

[131] Brackman LC Jung MS Green EH Joshi N Revetta FL McClain MS . IL-17 signaling protects against Helicobacter pylori-induced gastric cancer. Gut Microbes. (2024) 16:2430421. doi:10.1080/19490976.2024.2430421. PMID: 39588838 PMC11639209

[132] M H Y G X J L X K D K J . IL-17 signaling in steatotic hepatocytes and macrophages promotes hepatocellular carcinoma in alcohol-related liver disease. J Hepatol. (2020) 72(5):946–59. doi:10.1016/j.jhep.2019.12.016. PMID: 31899206 PMC7167339

[133] Denk D Ramakrishnan M Conche C Pallangyo C Pesic M Ceteci F . IL-17RA signaling provides dual tumor-suppressor function during late-stage colorectal carcinogenesis. Immunity. (2025) 58:701–715.e8. doi:10.1016/j.immuni.2025.02.005. PMID: 40023157

[134] S O N M A Y B J C E F R . Signaling controversy and future therapeutical perspectives of targeting sphingolipid network in cancer immune editing and resistance to tumor necrosis factor-α immunotherapy. Cell Communication Signaling: CCS. (2024) 22(1):251. doi:10.1186/s12964-024-01626-6. PMID: 38698424 PMC11064425

[135] Ababneh O Nishizaki D Kato S Kurzrock R . Tumor necrosis factor superfamily signaling: life and death in cancer. Cancer Metastasis Rev. (2024) 43:1137–63. doi:10.1007/s10555-024-10206-6. PMID: 39363128 PMC11554763

[136] Graier T Lwin SM Griffiths CEM . Breaking the cycle of psoriasis: the role for early intervention. Br J Dermatol. (2026) 194:359–61. doi:10.1093/bjd/ljaf403. PMID: 41123483

[137] Kimball AB Jemec GBE Sayed CJ Kirby JS Prens E Ingram JR . Efficacy and safety of bimekizumab in patients with moderate-to-severe hidradenitis suppurativa (BE HEARD I and BE HEARD II): two 48-week, randomised, double-blind, placebo-controlled, multicentre phase 3 trials. Lancet. (2024) 403:2504–19. doi:10.1016/S0140-6736(24)00101-6. PMID: 38795716

[138] Reich K Warren RB Lebwohl M Gooderham M Strober B Langley RG . Bimekizumab versus secukinumab in plaque psoriasis. N Engl J Med. (2021) 385:142–52. doi:10.1056/NEJMoa2102383. PMID: 33891380

[139] Warren RB Blauvelt A Bagel J Papp KA Yamauchi P Armstrong A . Bimekizumab versus adalimumab in plaque psoriasis. N Engl J Med. (2021) 385:130–41. doi:10.1056/NEJMoa2102388. PMID: 33891379

[140] Liu Y Wang H Taylor M Cook C Martínez-Berdeja A North JP . Classification of human chronic inflammatory skin disease based on single-cell immune profiling. Sci Immunol. (2022) 7:eabl9165. doi:10.1126/sciimmunol.abl9165. PMID: 35427179 PMC9301819

[141] Tang H Wang J Zhang S Feng G Cheng X Meng X . Housekeeping gene dysregulation in psoriasis: integrative multi-cohort and single-cell analysis reveals keratinocyte-centric molecular mechanisms and diagnostic biomarkers. Front Immunol. (2025) 16:1601705. doi:10.3389/fimmu.2025.1601705. PMID: 40959079 PMC12433973

[142] Z F S C H Y G J D J K R . DNA methylation-based subclassification of psoriasis in the Chinese Han population. Front Med. (2018) 12. doi:10.1007/s11684-017-0588-6. PMID: 29623515

[143] Nan T Zheng S Qiao S Quan H Gao X Niu J . Deep learning quantifies pathologists’ visual patterns for whole slide image diagnosis. Nat Commun. (2025) 16:5493. doi:10.1038/s41467-025-60307-1. PMID: 40595468 PMC12214614

[144] Huang C-H Lichtarge S Fernandez D . Integrative whole slide image and spatial transcriptomics analysis with QuST and QuPath. NPJ Precis Oncol. (2025) 9:70. doi:10.1038/s41698-025-00841-9. PMID: 40075141 PMC11904241

[145] . 146. Spatial transcriptomics inferred from pathology whole-slide images links tumor heterogeneity to survival in breast and lung cancer. PMID: [Accessed April 17, 2026]. 33139755 10.1038/s41598-020-75708-z PMC7606448

[146] Strober B Thaçi D Sofen H Kircik L Gordon KB Foley P . Deucravacitinib versus placebo and apremilast in moderate to severe plaque psoriasis: efficacy and safety results from the 52-week, randomized, double-blinded, phase 3 Program fOr Evaluation of TYK2 inhibitor psoriasis second trial. J Am Acad Dermatol. (2023) 88:40–51. doi:10.1016/j.jaad.2022.08.061. PMID: 36115523

[147] Morand E Pike M Merrill JT van Vollenhoven R Werth VP Hobar C . Deucravacitinib, a tyrosine kinase 2 inhibitor, in systemic lupus erythematosus: a phase II, randomized, double-blind, placebo-controlled trial. Arthritis Rheumatol. (2023) 75:242–52. doi:10.1002/art.42391. PMID: 36369798 PMC10100399

[148] Armstrong AW Gooderham M Warren RB Papp KA Strober B Thaçi D . Deucravacitinib versus placebo and apremilast in moderate to severe plaque psoriasis: efficacy and safety results from the 52-week, randomized, double-blinded, placebo-controlled phase 3 POETYK PSO-1 trial. J Am Acad Dermatol. (2023) 88:29–39. doi:10.1016/j.jaad.2022.07.002. PMID: 35820547

[149] Nadeem A Ahmad SF Al-Harbi NO El-Sherbeeny AM Alasmari AF Alanazi WA . Bruton’s tyrosine kinase inhibitor suppresses imiquimod-induced psoriasis-like inflammation in mice through regulation of IL-23/IL-17A in innate immune cells. Int Immunopharmacol. (2020) 80:106215. doi:10.1016/j.intimp.2020.106215. PMID: 31982823

